# Assessment of the measurement properties of the post stroke motor function instruments available in Brazil: a systematic review

**DOI:** 10.1590/bjpt-rbf.2014.0144

**Published:** 2016-03-15

**Authors:** Elaine Lima, Luci F. Teixeira-Salmela, Luan Simões, Ana C. C. Guerra, Andrea Lemos

**Affiliations:** 1 Departamento de Fisioterapia, Universidade Federal de Pernambuco (UFPE), Recife, PE, Brazil; 2 Departamento de Educação Física, Universidade Federal de Minas Gerais (UFMG), Belo Horizonte, MG, Brazil

**Keywords:** stroke, validity of tests, reproducibility of results, translating, physical therapy

## Abstract

**Background:**

While there are several instruments in Brazil that measure motor function in patients after stroke, it is unknown whether the measurement properties of these instruments are appropriate.

**Objective:**

To identify the motor function instruments available in Brazil for patients after stroke. To assess the methodological quality of the studies and the results related to the measurement properties of these instruments.

**Method:**

Two independent reviewers conducted searches on PubMed, LILACS, CINAHL, Web of Science, and Scopus. Studies that aimed to cross-culturally adapt an existing instrument or create a Brazilian instrument and test at least one measurement property related to motor function in patients after stroke were included. The methodological quality of these studies was checked by the COSMIN checklist with 4-point rating scale and the results of the measurement properties were analyzed by the criteria developed by Terwee et al.

**Results:**

A total of 11 instruments were considered eligible, none of which were created in Brazil. The process of cross-cultural adaptation was inadequate in 10 out of 11 instruments due to the lack of back-translation or due to inappropriate target population. All of the instruments presented flaws in the measurement properties, especially reliability, internal consistency, and construct validity.

**Conclusion:**

The flaws observed in both cross-cultural adaptation process and testing measurement properties make the results inconclusive on the validity of the available instruments. Adequate procedures of cross-cultural adaptation and measurement properties of these instruments are strongly needed.

## BULLET POINTS

- 11 studies were found that assessed post-stroke motor function in Brazilian patients.
- Most of the cross-cultural adaptation was conducted without the target population.
- Flaws in the measurement properties made the results inconclusive.
- Caution should be taken in the selection of instruments for research and clinical practice.

## Introduction

Various measurement instruments have been created with the objective of assessing motor function in post-stroke individuals^1^^-^ 3. These instruments aim to verify the ability to maintain or change the body's position in space, walk and move around, move and handle objects, as well as verify motor coordination and fine manual motricity^1^^-^ 3. These abilities involve aspects related to activities and participation and the structure and function of the organs and systems, as described in the International Classification of Functioning, Disability, and Health (ICF)^4^.

The application of these instruments aims to measure upper limb function, trunk function, or global motor function^1^^-^ 3. Some instruments assess performance through the observation of performed activities, while others are based on questionnaires on motor function^1^^-^ 3. After stroke, motor function can present various degrees of impairment and generate social and economic loss. Therefore, it is essential to use valid instruments to achieve an effective rehabilitation^5^^-^ 7.

In general, the instruments used in Brazil to assess post-stroke motor function were developed in other countries, usually in English and, consequently, targeted to the original population^1^^,^ 8. However, before an instrument can be used in a new country, culture, and/or language, a cross-cultural adaptation process is necessary. This process requires a standardized method involving the language translation and the cross-cultural adaptation to maintain its content validity^9^^,^ 10. After this process, the new scale should be applied to the new target population and its measurement properties can be analyzed to check if the adapted instrument truly measures the construct in the new setting^9^^-^ 12.

The instrument can only be considered valid and reliable for use in a new cultural-clinical context through the adequate evaluation of the measurement properties^9^^-^ 11. The objectives of this systematic review were to identify the measurement instruments of motor functions in post-stroke individuals available in Brazil, to assess the methodological quality of the studies, and to assess the results of these studies.

## Method

Two independent reviewers (EL and LS) conducted searches and selected eligible studies in the PUBMED, LILACS, SCOPUS, CINAHL, and WEB OF SCIENCE databases between February and March of 2014, according to the search strategy presented in Table 1. There was no language restriction.

**Table 1.** Research strategies for each research database.

**DATABASE**	**RESEARCH STRATEGY**
MEDLINE (PUBMED)	(("Brazil " [Mesh]) OR Portuguese OR Brazilian) AND (("Stroke" [Mesh]) OR ("Paresis" [Mesh])) AND (("Questionnaires" [Mesh]) OR scale OR test OR performance based test) AND Sensitive search filter for measurement properties NOT Exclusion Filter *
LILACS	(Brazil OR Portuguese Brazilian OR) AND (Stroke OR Stroke OR paresis) AND (Trunk OR upper limbs OR lower limbs OR sensorimotor function OR motor OR Function motor activity OR mobility OR coordination balance OR instrumentation OR comparative studies OR validation studies OR translations OR translation adjustment OR cross-cultural equivalence OR Validity OR validation OR Reliability OR reproducibility OR reproducible OR psychometric tests OR psychometric properties OR clinimetric clinimetric OR property OR valuation OR inter-observer OR variation results OR Intra-examiner OR mony retest OR inter-rater OR intraobserver OR interparticipants OR intraparticipants OR internal consistency Rasch OR Effect OR Effect floor ceiling OR disability assessment OR questionnaires OR scale tests)
CINAHL AND WEB OF SCIENCE	(Brazil OR Brazilian OR Portuguese) AND (Stroke OR Paresis) AND (questionnaires OR scale OR test OR comparative studies OR validation studies OR validation OR translations OR cross cultural OR cross cultural adaptation OR cross cultural comparison OR cross-cultural equivalence OR validity OR reliability OR reproducibility OR psychometrics OR clinimetrics OR outcome assessment OR observer variation OR reproducibility of results OR internal consistency OR alpha Cronbach OR agreement OR precision OR test retest OR interrater OR inter-rater OR intrarater OR intra-rater OR intertester OR inter-tester OR intratester OR intra tester OR interobserver OR inter-observer OR intraobserver OR intraobserver OR intertechnician OR inter-technician OR intratechnician OR intra-technician OR interexaminer OR inter examiner OR intraexaminer OR intra-examiner OR interassay OR inter-assay OR intraassay OR intra-assay OR interindividual OR inter individual OR intraindividual OR intra-individual OR interparticipant OR inter-participant OR intraparticipant OR intra-participant OR kappa OR concordance OR intraclass OR dimension OR subscale OR responsivity OR ceiling effect OR ﬂoor effect OR Item response model OR IRT OR Rasch)
SCOPUS	TITLE-ABS-KEY(stroke OR paresis) AND TITLE-ABS-KEY(cross cultural adaptation OR cross cultural comparison OR translation) AND TITLE-ABS-KEY(Brazil OR Brazilian OR Portuguese)
	TITLE-ABS-KEY(stroke OR paresis) AND TITLE-ABS-KEY(validation OR validity OR validation studies) AND TITLE-ABS-KEY(Brazil OR Brazilian OR Portuguese)
	TITLE-ABS-KEY(stroke OR paresis) AND TITLE-ABS-KEY(psychometrics OR clinimetrics OR reliability OR reproducibility OR responsiveness OR internal consistency OR intra examiner OR inter examiner OR responsivity OR ceiling effect OR floor effect) AND TITLE-ABS-KEY(brazil OR brazilian OR portuguese)

*In accordance with the recommendations of the Consensus-based Standards for the selection of health Measurements Instruments-COSMIN^13^.

Either cross-cultural adaptation studies or Brazilian instruments that assessed the motor function of post stroke individuals in at least one item were considered eligible. Furthermore, these studies had to have verified at least one measurement property of these instruments. Studies that involved individuals with other neurological conditions were excluded.

The two reviewers (EL and LS) screened the studies by title and abstract performing a pre-selection through eligibility criteria on the computer screen. Then, they read the full text of the studies potentially eligible to confirm their inclusion. It was pre-defined that disagreements between two reviewers were arbitrated by a third reviewer (AL).

The data extraction was performed in a standardized way through a pre-established data extraction form. The following data were extracted: title, authors, year of publication, journal, study objectives, eligibility criteria of the participants, instrument objective (discriminative, predictive or evaluative)^12^, number of subscales/items/domains, and domain assessed according to ICF^4^.

The evaluation of the methodological quality of the included studies was performed through the COSMIN checklist with 4-point rating scale, which is a tool created through the Consensus-based Standards for the selection of health Measurements Instruments (COSMIN)*,* with the aim of scoring and classifying the quality of the methods used for the study of each measurement property^14^^-^ 16.

The COSMIN checklist with 4-point rating scale is composed of nine boxes: A- Internal consistency, B- Reliability, C- Measurement error, D- Content validity, E- Structural validity, F- Hypothesis tests, G- Cross-cultural validity, H- Criterion validity, and I- Responsiveness^14^^-^ 16.

Each box includes a series of items that assess the measurement property methodology. These items are classified on a scale of 4 points: 1- Poor, 2- Fair, 3- Good, and 4- Excellent. The final classification for each box is determined by the lowest score achieved by any of the items^14^^-^ 16.

In addition to the boxes mentioned above, there is still another box that should be completed for each measurement property. This box aims to identify the clinical-epidemiological profile of the population, analyzing age mean, distribution by gender, illness characteristic, country of origin, and spoken language^14^^-^ 16.

For example, to assess the internal consistency, box A presents 11 items: the first 3 items assess the missing data. Item 4 assesses the sample size; items 5, 6, and 7 assess questions related to unidimensionality; item 8 verifies the presence of other methodological flaws; and the other items verify the statistical method^14^^-^ 16.

Item 4 of this box assesses the sample size for internal consistency as follows: Excellent (N=100), Good (between 50 and 99 participants), Fair (between 30 and 40 participants), and Poor (less than 30 participants). Similar to item 4, the remaining items are scored on a 4-point scale, according to specific criteria. In the end, even if the instrument has obtained "excellent" classification in the other items, but in item 4 received a "good" score for having a sample size between 50 and 99 participants, the internal consistency of the instrument will be classified as having "good" internal consistency as the lowest score is used^14^^-^ 16.

Furthermore, the COSMIN recommends that to complement the evaluation of an instrument, the quality criteria developed by Terwee et al.^11^ should be used; these criteria classify the measurement properties as Positive (+), Negative (-), or doubtful (?) focusing on the analysis of the obtained results^11^. The use of the Terwee et al.^11^ criteria complements the evaluation of the measurement properties, as the COSMIN does not determine the cut offs that are considered adequate for the statistical analysis of each measurement property. In other words, the fact that a study used Cronbach's α, one of the statistical measurements advocated by COSMIN, to verify the internal consistency does not guarantee the quality of this property, as adequate values may not have been reached^11^^,^ 14 ^-^ 16.

For example, internal consistency receives a positive score when the unidimensionality is verified, with the participation of 100 or more individuals and through Cronbach's α (between 0.70 and 0.95). If α does not reach this interval, the score will be negative. When the unidimensionality is not verified, or if there is another methodological flaw, the score will be classified as inconclusive^11^.

## Results

**Figure 1.**
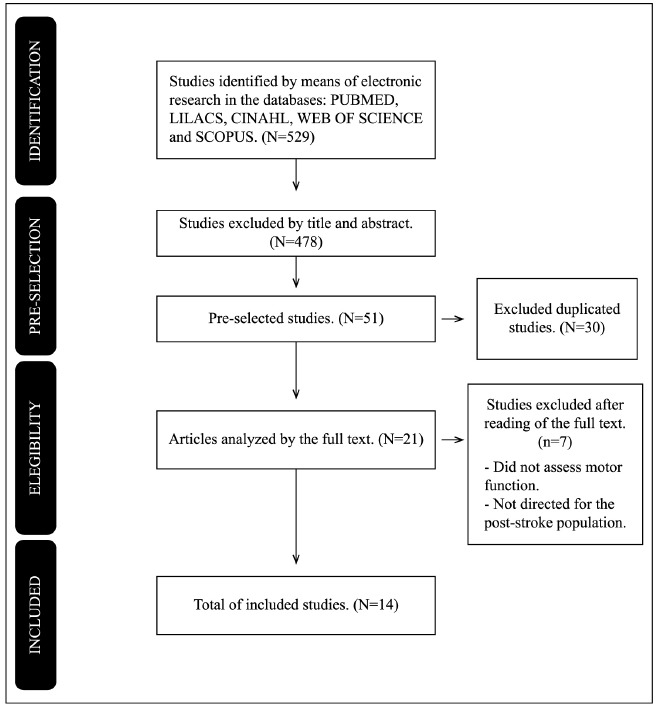
Identification, selection, and inclusion of the studies.

A total of 529 studies were found, of which only 14 studies^17^^-^ 30 were included through the eligibility criteria (Figure 1). Two instruments (Test Évaluant les Membres supérieurs des Personnes Âgées - TEMPA^20^ and the Jebsen-Taylor Test^21^) were not specifically created for post-stroke individuals; however, they have been validated in Brazil for this population and were included in this review.

In the 14 studies included, 11 instruments were identified. Three of them (Motor Activity Log - MAL^18^^,^ 19, Fugl-Meyer scale^23^^,^ 24, and National Institute of Health Stroke Scale - NIHSS^25^^,^ 26) were analyzed in two studies each, and the other 8 in only one study. The characterization of the included studies is presented in Table 2.

**Table 2.** Description of the instruments that assess the motor function of post-stroke individuals available in Brazil.

**INSTRUMENT**	**AUTHOR/YEAR**	**CONSTRUCT**	**OBJECTIVE**	**TYPE OF INSTRUMENT**	**DIMENSION OF CIF**	**Nº OF ITEMS/ SUBSCALES**	**BRAZIL VERSION (AUTHOR/YEAR)**
Postural Assessment Scale	Benaim et al.^31^ (1999)	Maintenance and postural changes	Evaluative	Based on performance	Activity	12 items	Yoneiama et al.^28^ (2008)
Trunk impairment Scale	Fujiwara et al.^32^ (2004)	Trunk motor function	Evaluative	Based on performance	Structure and function	7 items	Lima et al.^30^ (2008)
Trunk impairment Scale	Verheyden et al.^33^ (2004)	Trunk motor function	Evaluative	Based on performance	Structure and function /Activity	17 items	Castelassi et al.^29^ (2009)
Fugl-Meyer Scale	Fugl-Meyer et al.^34^ (1975)	Global sensory-motor function	Evaluative	Based on performance	Structure and function /Activity	7 subscales	Maki et al.^24^ (2006) Michaelsen et al.^23^ (2011)
Jebsen-Taylor Test	Jebsen et al.^35^ (1969)	Upper limb motor function	Evaluative	Based on performance	Activity	7 items	Ferreiro et al.^21^ (2010)
Motor Assessment Scale (MAS)	Carr et al.^36^ (1985)	Global motor function	Evaluative	Based on performance	Activity	8 items	Conte et al.^27^ (2009)
Motor Activity Log (MAL)	Uswatte et al.^37^ (2006)	Upper limb motor function	Evaluative	Questionnaire	Participation	30 items	Saliba et al.^18^ (2011) Pereira et al.^19^ (2012)
National Institutes of Health stroke scale (NIHSS)	Brott et al.^38^ (1989)	Losses from the Stroke	Predictive	Based on performance	Activity	11 items (2 for motor function)	Cincura et al.^25^ (2009) Caneda et al.^26^ (2006)
Rivermead Mobility Index (RMI)	Collen et al.^39^ (1991)	Global motor function	Evaluative	Questionnaire	Activity	15 items	Pavan et al.^22^ (2010)
TEMPA (Test Évaluant les Membres supérieurs des Personnes Âgées)	Desrosiers et al.^40^ (1993)	Upper limb motor function	Evaluative	Based on performance	Activity	9 items (8 items in the Brazilian version)	Michaelsen et al.^20^ (2008)
Wolf Motor Function Test	Morris et al.^41^ (2004)	Upper limb motor function	Evaluative	Based on performance	Structure and function /Activity	17 items	Pereira et al.^17^ (2011)

None of the included instruments were Brazilian, therefore all had to be submitted to the cross-cultural adaptation process. The most frequently measured properties were reliability (n=11 studies), construct validity through hypothesis testing (n=6 studies), and internal consistency (n=6 studies). None of the included studies assessed responsiveness. The evaluation of the measurement properties is shown in Table 3.

**Table 3.** Evaluation of the methodological quality of the included studies through the COSMIN checklist with 4-point rating scale: Consensus-based standard for the selection of health measurement instruments.

**INSTRUMENT**	**AUTHOR/YEAR**	**USED TRI**	**TRI**	**INTERNAL CONSISTENCY (A)**	**RELIABILITY (B)**	**MEASUREMENT ERROR (C)**	**F HYPOTHESIS TEST (F)**	**CROSS-CULTURAL ADAPTATION (G)**	**GENERALIZATION BY BOX**
Postural Assessment Scale	Yoneiama et al.^28^ (2008)	No	-	Poor	(Inter and test-retest) Poor	-	Poor	Fair	A Excellent B Excellent C Excellent F Excellent G Poor
Trunk Commitment Scale	Lima et al.^30^ (2008)	No		Poor	(Inter and test-retest) Poor	-	Poor	Poor	A Excellent B Excellent F Excellent G Poor
Trunk Deficiency Scale	Castelassi et al.^29^ (2009)	No	-	Poor	Poor	Poor	Poor	Fair	A Excellent B Excellent F Excellent G Reasonable
Fugl-Meyer Scale	Maki et al.^24^ (2006)	No	-	-	(Inter and test-retest) Poor	-	-	Fair	B Excellent G Poor
Fugl-Meyer Scale (manual)	Michaelsen et al.^23^ (2011)	No	-	-	(Inter) Poor	-	-	Poor	B Excellent G Poor
Jebsen-Taylor Test	Ferreiro et al.^21^ (2010)	No	-	Poor	(Inter and Intra) Fair	-	-	Poor	A Excellent B Excellent G Poor
Motor Assessment Scale	Conte et al.^27^ (2009)	No	-	-	(Inter and Intra) Poor	-	-	Poor	B Excellent G Poor
Motor Activity Log	Saliba et al.^18^ (2011)	Yes	Good	Poor	Poor (test-retest)	-	-	Excellent	A Excellent B Excellent F Excellent G Excellent
Motor Activity Log	Pereira et al.^19^ (2012)	No	-	-	(Inter and test-retest) Poor	(Inter and test-retest) Poor	Fair	-	B Excellent C Excellent F Excellent
National Institute of Health stroke scale	Cincura et al.^25^ (2009)	No	-	-	Good	-	Fair	Poor	B Excellent F Excellent G Poor
National Institute of Health stroke scale	Caneda et al.^26^ (2006)	No	-	-	Good	-	-	Poor	B Excellent
Rivermead Mobility Index	Pavan et al.^22^ (2010)	No	-	Poor	Poor (test-retest)	-	-	Good	A Excellent B Excellent G Poor
TEMPA	Michaelsen et al.^20^ (2008)	No	-	-	(Inter and test-retest) Poor	-	Poor	Poor	B Excellent F Excellent G Poor
Wolf Motor Function Test	Pereira et al.^17^ (2011)	No	-	-	(Inter and Intra) Poor	(Inter and Intra) Poor	-	Poor	B Excellent C Excellent G Poor

TRI: Theory of the Answer to the TEMPA item: Test Évaluant les Membres supérieurs des Personnes Âgées. The following properties were not analyzed in the studies: D: Content Validity; E: Structural validity; H: Criteria validity; I: Responsiveness; J: Interpretability.

### Cross-cultural adaptation

Only one instrument (MAL)^18^^,^ 19 was adapted to Brazilian culture in accordance with the recommended method^9^^,^ 14 ^-^ 16. The cross-cultural adaptation of the Rivermead Mobility Index (RMI)^22^ obtained a "good" classification, given that it is not clear whether an expert committee participated or whether a pre-test was conducted^9^.

Six instruments^17^^,^ 21 ^,^ 23 ^,^ 25 ^-^ 27 ^,^ 30 obtained a classification considered "poor" for the cross-cultural adaptation process. The TEMPA^20^ and Motor Assessment Scale (MAS)^27^ were created through simple translations into Portuguese. The NIHSS^25^^,^ 26 was inadequately adapted in two studies, one of which only performed a single translation into Portuguese^25^. In the adaptation of the Wolf Motor Test (Wolf)^17^, Jebsen-Taylor Test^21^, and the Trunk Impairment Scale^30^, pre-tests were not performed either.

For the Fugl-Meyer Scale^23^^,^ 24, Posture Assessment Scale^28^, and Trunk Deficiency Scale^29^, the quality of the adaptation process was "fair" because it included a pre-test but did not include an adequate description of the assessed sample. However, the Fugl-Meyer Scale^23^ manual, which was produced in a different study to the production of the instrument, presented a "poor" process as it included only one translation into Portuguese. In the Posture Assessment Scale^28^ and Trunk impairment Scale^29^, the translation and back translation were performed by only one translator.

### Reliability

All of the instruments were tested for reliability. Eight (MAS^27^, MAL^18^^,^ 19, Wolf Motor Function Test^17^, TEMPA^20^, Posture Assessment Scale^28^, Trunk impairment Scale^30^, and the study of the Fugl-Meyer manual^24^) received a "poor" classification because they included fewer than 30 participants and used the intraclass correlation coefficient (ICC) when this was not indicated. The studies of the Fugl-Meyer Scale^23^ (N=50) and RMI^22^ (N=95) had good samples but were classified as "poor" for having used inadequate statistical methods (i.e. ICC and the Wilcoxon test, respectively). The Jebsen-Taylor Test^21^ was considered "fair" for presenting a sample between 30 and 49 individuals (n=40). The reliability of the NIHSS was verified in two studies with "good" methodology and samples of 51 and 62 participants, respectively^25^^,^ 26.

### Measurement error

The measurement error was verified in three instruments (Trunk impairment Scale^29^, Wolf Motor Function Test^17^, and MAL^18^^,^ 19) through the Bland Altman plot analysis; however, the methodological quality was classified as "poor" because the sample included less than 30 individuals.

### Internal consistency

Six instruments^18^^,^ 19 ^,^ 21 ^,^ 22 ^,^ 28 ^-^ 30 were tested for internal consistency; however, the methodological quality was classified as "poor" in all of them. In five instruments (Posture Assessment Scale^28^, RMI^22^, Jebsen Taylor Test^21^, and Trunk impairment Scales^29^^,^ 30), the reason was the lack of factor analysis. Moreover, in the Posture Assessment Scale^28^ and Trunk impairment Scales^29^^,^ 30, the sample included less than 30 individuals and in the study of MAL^18^^,^ 19, the sample included less than 5 individuals per item of the instrument for unidimensionality.

### Construct validity

Construct validity was analyzed in six instruments (MAL^19^, TEMPA^20^, Posture Assessment Scale^28^, NIHSS^25^, and Trunk Impairment Scales^29^^,^ 30) through the hypothesis tests by correlation with the Fugl Meyer Scale^19^^,^ 20 ^,^ 28 ^,^ 29, Barthel Index^25^, Berg Balance Scale, and Functional Independence Measure^30^. The study method used in four of these instruments was classified as "poor" due to inadequate sample size (n<30)^20^^,^ 28 ^-^ 30.

The MAL^19^ and NIHSS^25^^,^ 26 presented "fair" methodological quality in the validity tests, as the hypotheses about the direction and magnitude of the correlation were not previously formulated or described in the study; however, it was possible to assume the expected direction for the correlation (positive or negative).

### Terwee criteria

As for the evaluation of the results of the measurement property analysis using the criteria of Terwee et al.^11^, the majority of the studies presented doubtful results in the study of measurement properties, with the exception of the inter-examiner reliability of the NIHSS^25^^,^ 26, which presented positive results with Kappa coefficient >0.70 in items 5a, 5b, 6a, and 6b (referring to upper and lower limb motor function) (Table 4).

**Table 4.** Measurement properties assessment through the Terwee et al.

**Instrument**	**Author/ year**	**Internal Consistency**	**Reliability**	**Measurement error**	**Content validity**	**Construct validity**	**Criteria validity**	**Responsiveness**	**Floor and ceiling effect**	**Interpretability**
Posture Assessment Scale	Yoneima et al.^28^ (2008)	?	(inter and test-retest) ?	NA	NA	?	NA	NA	?	NA
Trunk commitment Scale	Lima et al.^30^ (2008)	?	?	NA	NA	?	NA	NA	NA	NA
Trunk Deficiency Scale	Castelassi et al.^29^ (2009)	?	?	?	NA	?	NA	NA	?	NA
Fugl-Meyer Scale	Maki et al.^24^ (2006)	NA	(Inter and test-retest) ?	NA	NA	NA	NA	NA	NA	NA
Fugl-Meyer Scale Manual	Michaelsen et al.^23^ (2011)	NA	(Inter) ?	NA	NA	NA	NA	NA	NA	NA
Teste de Jebsen-Taylor	Ferreiro et al.^21^ (2010)	?	(inter and intra) ?	NA	NA	NA	NA	NA	NA	NA
Motor Assessment Scale	Conte et al.^27^ (2009)	NA	(inter and intra) ?	NA	NA	NA	NA	NA	NA	NA
Motor Activity Log	Saliba et al.^18^ (2011)	?	(test-retest) ?	NA	NA	?	NA	NA	NA	NA
Motor Activity Log	Pereira et al.^19^ (2012)	NA	(inter and test-retest) ?	(inter and test-retest) ?	NA	?	NA	NA	NA	NA
NIHSS	Cincura et al.^25^ (2009)	NA	(inter) +	NA	NA	?	NA	NA	NA	NA
NIHSS	Caneda et al.^26^ (2006)	NA	(inter) +	NA	NA	NA	NA	NA	NA	NA
Rivermead Mobility Index	Pavan et al.^22^ (2010)	?	(test-retest) ?	NA	NA	NA	NA	NA	NA	NA
TEMPA	Michaelsen et al.^20^ (2008)	NA	(inter and test-retest) ?	NA	NA	?	NA	NA	NA	NA
Wolf Motor Function Test	Pereira et al.^17^ (2011)	NA	(Inter and intra) ?	(Inter and intra) ?	NA	NA	NA	NA	NA	NA

(+)= Positive; (-)= Negative; (?)= Inconclusive; (NA)= Not Assessed. TEMPA: Test Évaluant les Membres supérieurs des Personnes Âgées; NIHSS: National Institute of Health Stroke Scale.

The results of the measurement error tests of the internal consistency and of construct validity were considered doubtful due to the methodological flaws presented, as described previously^17^^,^ 19 ^,^ 28.

The ceiling and floor effects, which reflect interpretability, were verified in two instruments (Trunk Impairment Scale^29^ and Posture Assessment Scale^28^). The percentage of individuals who reached the minimum and maximum scores was lower than 15%, but with an inadequate sample size (<50). However, other measures of interpretability like the minimum clinically important difference and minimum important difference were not analyzed. Finally, criterion validity and responsiveness were not tested in any of the eligible studies.

## Discussion

The results of this review showed that the available instruments in Brazil for assessing post-stroke motor function are arising from cross-cultural adaptation, not from newly developed Brazilian. However, the findings are inconclusive regarding the quality of the cross-cultural adaptation as well as from measurement properties, due to flaws with regards to methodology. The main methodological flaw observed during the cross-cultural adaptation process of the included instruments was the absence of a pre-testing of the final version^17^^,^ 20 ^-^ 22 ^,^ 27 ^,^ 30. Only one instrument (Motor Activity Log - MAL)^18^^,^ 19 followed the recommended processes for an adequate cross-cultural adaptation.

The goal of applying the instrument in the target population (pre-test) before the measurement property analysis aims to identify possible imperfections in the interpretation of the items of an interview and the viability of the tasks proposed by the instrument for the target population. Therefore, the performance of the pre-test allows the identification of possible adjustments necessary in the instrument, based on the direct participation of the population for which it was adapted^11^.

Although some instruments performed a pre-test, most of the studies did not described the sample properly^24^^,^ 28 ^,^ 29. To allow the generalization of the results of a cross-cultural adaptation, the COSMIN checklist recommends that the participants involved in the pre-test should be clinically and epidemiologically reported in terms of age, gender, characteristics of the illness, and source of patients (hospital, clinic, community, etc.)^14^^-^ 16.

The absence of a back translation was also verified in some instruments^20^^,^ 27. This stage has the important aspect of allowing the verification of semantic equivalence between the original instrument and what was created in the new language, allowing necessary adjustments in the new version. It was also observed that, in some instruments^28^^,^ 29, the stages of translation and back translation were performed by a single translator. The performance of multiple translations is recommended in the literature because it allows the interaction between specialists in the construct and in the languages involved, allowing a more adequate process of cultural adaptation and the maintenance of semantic equivalence^14^^-^ 16.

Concerning the measurement properties, methodological flaws were also verified. The reliability was verified in all studies; however, in the majority of these, a sample size of less than 30 participants was selected^17^^-^ 20 ^,^ 22 ^,^ 27 ^-^ 30. The adequate number is at least 50 participants, and for an ideal sample, the recruitment of at least 100 participants is recommended^14^^-^ 16.

In addition, the intra-lass correlation coefficient was often chosen as the statistical method when it was, in fact, inadequate. The adequate method for instruments with ordinal type scores is the Kappa coefficient^17^^-^ 20 ^,^ 22 ^-^ 24 ^,^ 27 ^-^ 30. The only instrument with an adequate study method for reliability, the NHSS, presented flaws in the cross-cultural adaptation^25^^,^ 26.

For internal consistency, the majority of the studies did not report factorial analysis or unidimensionality study of the items^21^^,^ 22 ^,^ 28 ^-^ 30. These analyses are important because they intend to verify the number of dimensions into which the items are distributed and whether subscales are formed in the instrument. The only instrument to present the unidimensionality through the Rasch analysis, the (MAL)^18^^,^ 19, included an inadequate number of participants^14^^-^ 16.

In terms of internal consistency, a sample of 7 participants is indicated for each item of the instrument, requiring a minimum of 100. For example, for an instrument of 30 items, a sample of 210 would be indicated^14^^-^ 16. It is recommended that internal consistency should be assessed in two ways: through the classic form, or by the item response theory. First, Cronbach's alpha should be calculated after the performance of the factorial analysis, which identifies the number of subscales where the alpha must be calculated^14^^-^ 16. Second, the Rasch mathematical model is indicated to assess the unidimensionality of the items, verifying the presence of items that can be adjusted or removed from the instrument^14^^-^ 16.

The flaws observed in the construct validity of the instruments^20^^,^ 25 ^,^ 26 ^,^ 28 ^-^ 30 generate uncertainties about the degree to which the Brazilian versions of the included instruments truly measure the correct construct. It is recommended that 100 participants be assessed and that hypotheses be previously formulated about the direction and magnitude of the expected correlation between the scores of the tested instruments and the comparator instrument^14^^-^ 16.The responsiveness and the criterion validity were not analyzed in any of the studies. The criterion validity is analyzed to verify the degree with which the scores of the instruments are an adequate reflection of the "gold-standard". However, for motor function measurement, no such instrument was observed in Brazil.

The absence of the responsiveness study, observed in all of the instruments, hampers the identification of the ability of these instruments to detect changes in the assessed construct over time. Therefore, there is no evidence that it will be possible to quantify any motor function changes in post-stroke individuals in clinical research^14^^-^ 16.

Finally, the interpretability of the obtained scores in these instruments still has not been clarified. Despite the fact that the ceiling and floor effects in the Posture Assessment Scale and Trunk Deficiency Scale were analyzed and had favorable results, the sample size in both studies was inadequate. None verified the minimum important change (MIC) or the minimum important difference (MID). These results are relevant because the MIC is the smallest change in the construct score the patients observe as important and the MID corresponds to the minimum difference in the construct among patients that is considered important^14^^-^ 16. None of the instruments were tested for their interpretability and responsiveness. As such, it remains unknown whether these instruments are able of measuring clinical changes over time.

## Final considerations

Future studies should revise the cross-cultural adaptation processes, following all of the recommended stages (translation, synthesis of translations, back translation, expert committee, and pre-test). Moreover, the measurement properties should be analyzed with an adequate number of participants and the application of statistical methods that reflect the validity of each property. The results of this review point out that health professionals must be cautious when selecting instruments to assess post-stroke motor function for use in research and clinical practice in Brazil.
